# Apricot Bee Pollen Alleviates Deoxynivalenol-Induced Cellular Toxicity in Bovine Granulosa Cells

**DOI:** 10.3390/ani15111580

**Published:** 2025-05-28

**Authors:** Ce Lv, Xiaoxue Zheng, Hanxiao Wu, Peihao Sun, Qun Lu, Fang Fang, Mingxiao Liu, Shuo Zhou, Rui Liu, Xiang Li, Liguo Yang, Aixin Liang

**Affiliations:** 1 Key Laboratory of Agricultural Animal Genetics, Breeding and Reproduction, Ministry of Education, College of Animal Science and Technology, Huazhong Agricultural University, Wuhan 430070, China; lvce829@163.com (C.L.); zhengxx@webmail.hzau.edu.cn (X.Z.); whxqwer920@163.com (H.W.); sph99@webmail.hzau.edu.cn (P.S.); fangfang@mail.hzau.edu.cn (F.F.); liumingxiao@webmail.hzau.edu.cn (M.L.); xiaolv@webmail.hzau.edu.cn (S.Z.); xxianglli@mail.hzau.edu.cn (X.L.); ylg@mail.hzau.edu.cn (L.Y.); 2 College of Food Science and Technology, Huazhong Agricultural University, Wuhan 430070, China; luqun@mail.hzau.edu.cn (Q.L.); liurui@mail.hzau.edu.cn (R.L.); 3 Frontiers Science Center for Animal Breeding and Sustainable Production, Ministry of Education, Huazhong Agricultural University, Wuhan 430070, China

**Keywords:** apricot bee pollen, deoxynivalenol, oxidative stress, steroidogenesis, granulosa cells

## Abstract

Apricot bee pollen is an important natural product that exhibits antioxidant, anti-inflammatory, and antimicrobial properties. Deoxynivalenol (DON), one of the most prevalent mycotoxins produced by *Fusarium* fungi, poses risks to both human and animal reproductive systems. We observed that exposure to DON inhibited cell proliferation and induced apoptosis in bovine granulosa cells (bGCs), accompanied by a significant downregulation of PCNA expression and an upregulation of BAX expression. RNA sequencing analysis revealed that differentially expressed genes (DEGs) were primarily enriched in the oxidation-reduction process, oxidoreductase activity, and steroid biosynthesis. We further confirmed that DON exposure inhibited the production of estrogen and progesterone by decreasing the protein expression levels of CYP19A1 and StAR. Additionally, DON exposure increased the levels of reactive oxygen species (ROS) and malondialdehyde (MDA) in a dose-dependent manner, suggesting that DON induced oxidative stress in bGCs. Importantly, we demonstrated that apricot bee pollen ethanol extract (ABPE) increased the cell viability of bGCs and alleviated the effects of DON-induced cell viability reduction and estrogen dysfunction. Furthermore, ABPE attenuated the DON-induced increase in ROS levels and upregulated the expression of antioxidant-related gene *heme oxygenase-1 (HO-1)*. These results reveal the protective effects of ABPE against DON-induced cell viability reduction, estrogen disorder, and oxidative stress, providing new insights into the potential of bee pollen as a promising natural agent to improve mycotoxin contamination.

## Simple Summary

In the present study, we explored the toxic effects and mechanisms of deoxynivalenol exposure in bovine granulosa cells, and observed that deoxynivalenol could induce apoptosis, estrogen dysfunction, and oxidative stress by increasing the levels of reactive oxygen species and malondialdehyde. Importantly, apricot bee pollen ethanol extract was found to mitigate the reduction in cell viability, restore estrogen balance, and alleviate oxidative stress in bovine granulosa cells induced by deoxynivalenol. Our study provides new insights regarding the protective effects of bee pollen against cellular toxicity induced by deoxynivalenol, suggesting that bee pollen is a promising natural agent for preventing reproductive toxicity caused by mycotoxin contamination.

## 1. Introduction

Deoxynivalenol (DON) is one of the most prevalent mycotoxins produced by *Fusarium* fungi and is harmful to both humans and animals. DON is commonly found in livestock feeds, such as corn silage, concentrations, and other feedstuffs [1,2]. In recent years, the contamination of DON has garnered increasing attention due to its potential to cause significant economic losses and certain safety issues [3,4]. Although cattle are reportedly less sensitive to DON than pigs and poultry, DON can still be detected in the blood serum of cows exposed to natural DON [5], suggesting that it is not completely degraded by the microorganisms in the digestive tract of cattle. Moreover, both DON and de-epoxy DON can be detected in the milk, urine, and feces of cows that have consumed contaminated feed [6,7].

Granulosa cells (GCs) are a type of ovarian somatic cell that plays a crucial role in follicular development, oocyte maturation, and ovulation. Previous studies have demonstrated that DON can inhibit cell proliferation, induce apoptosis, and disrupt steroidogenesis in ovarian GCs of mice, humans, cattle, and pigs [8,9,10,11]. Moreover, DON can directly disturb several enzymes involved in steroidogenesis, such as CYP11A1, CYP19A1, and StAR, potentially by binding to ribosomes and triggering a ribotoxic stress response via p38, ERK1/2, and c-Jun [12]. In addition, Fan et al. [13] reported that DON exposure increased intracellular reactive oxygen species (ROS) levels, reduced mitochondrial membrane potential, and decreased ATP levels in murine GCs. Given the significant toxicity of DON in ovarian granulosa cells, several studies have attempted to ameliorate the ovarian toxicity induced by DON. Melatonin has been proven to alleviate DON-induced oxidative stress in murine GCs [13] and apoptosis in human GCs [9]. Besides melatonin, resveratrol and amygdalin have also been reported to alleviate DON-induced dysregulation of steroid hormones in porcine GCs [14,15]. However, there are few reports on alleviating the toxic effects of DON in bovine ovarian GCs.

Bee pollen (BP) is a natural health product derived from the pollen of flowering plants, combined with the bee digestive secretion enzymes and nectar [16]. It has been proven that BP possesses anti-inflammatory, antimicrobial, antifungicidal, and antioxidant properties [17,18], and it is utilized as both a medical agent and a nutritional supplement in China. In livestock, BP has been shown to enhance growth performance and immune responses [16]. Notably, El-Bialy et al. [19] demonstrated that bee pollen exerts a protective effect against certain immunotoxic effects caused by aflatoxin consumption. Additionally, it has been reported that the ethanol extract from BP is rich in polyphenols and exhibits significant anti-inflammatory activity in rats [20]. As a common kind of bee pollen, apricot bee pollen is rich in phenolamides, which can serve as a functional dietary supplement to alleviate obesity induced by a high-fat diet [21]. However, it remains unclear whether bee pollen, particularly apricot bee pollen, provides protection against DON-induced toxicity.

In the present study, we aimed to evaluate the toxic effects and mechanisms of DON exposure on bovine GCs, and to explore the potential protective effects of apricot bee pollen against DON-induced ovarian toxicity. These findings are crucial for developing strategies to mitigate reproductive toxicity in livestock.

## 2. Materials and Methods

### 2.1. Ethics Statement

All animal experiments in this study were approved by the Scientific Ethics Committee of Huazhong Agricultural University (HZAUCA-2022-0001) and were conducted in accordance with the Laboratory Animal Care and Use Guidelines of the Research Ethics Committee of Huazhong Agricultural University.

### 2.2. Reagents

Apricot bee pollen ethanol extract (ABPE) was provided by Professor Rui Liu’s team at the College of Food Science and Technology, Huazhong Agricultural University. DON (Sigma-Aldrich, St. Louis, MO, USA) was dissolved in absolute ethanol, and subsequently diluted with the medium. The final concentrations of DON were 0.1, 0.5, and 1 μmol/L, with the ethanol concentration remaining below 0.1% [22].

### 2.3. Measurement of Total Phenolics

The total phenolic content of the extracts was measured using the Folin–Ciocalteu method, as described by Liberato et al. [23], with minor modifications. Briefly, a dilute solution of apricot bee pollen in absolute ethanol (absolute ethanol–apricot bee pollen; 200 μL of 200 μg/mL) was mixed with 1 mL of Folin–Ciocalteu reagent and 1.5 mL of Na_2_CO_3_ (20% *w*/*v*). After incubation at room temperature (RT) in the dark for 1 h, the absorbance of the reaction mixture at 760 nm was measured using a microplate reader (PerkinElmer, Waltham, MA, USA). Gallic acid standard solutions were used to construct the calibration curve (y = 12.703x − 0.2494; R^2^ = 0.9971). The total phenols content was expressed as milligram (mg) of gallic acid equivalents (GAEs) per gram (g) of bee pollen.

### 2.4. Cell Culture

The ovaries were collected from healthy cows at a local slaughterhouse (Wuhan, Hubei, China) and transported to the laboratory in phosphate-buffered saline (PBS) supplemented with a 4% penicillin–streptomycin solution (Biosharp, Hefei, China) in a 37 °C incubator. Afterward, the ovaries were washed with PBS containing 4% penicillin–streptomycin solution and sterilized with 75% alcohol to fully wash. GCs were isolated from medium-small follicles (2–9.9 mm in diameter) using a 25-gauge needle aspiration, and filtered through 40 µm cell strainers (Biosharp, Hefei, China) to remove the oocytes. The GCs and follicular fluid were separated by centrifugation at 350× *g* for 10 min. Following this, the GCs were subjected to two washing cycles using DMEM/high glucose medium supplemented with 2% penicillin–streptomycin solution. The washed cells were then seeded into cell culture plates containing DMEM/high glucose medium enriched with 10% fetal bovine serum (FBS; Gibco, Carlsbad, CA, USA) and 1% penicillin–streptomycin solution. The cells were cultured in a humidified incubator under standardized conditions of 37 °C and 5% CO_2_ atmosphere.

### 2.5. Cell Viability Assay

Cell viability was assessed using the Cell Counting Kit-8 (CCK-8; Dojindo, Kumamoto, Japan) according to the manufacturer’s protocol. Specifically, cells were plated in 96-well plates and subsequently exposed to either DON (0, 0.1, 0.5, or 1 μmol/L) or ABPE (100, 200, and 300 μg/mL) for 24 h. Then, the cells were washed and 90 μL of FBS-free medium along with 10 μL of CCK-8 were added to each well. The 96-well plates were then incubated in the dark at 37 °C for 2 h. Finally, the absorbance was measured at 450 nm using a microplate reader.

### 2.6. Cell Cycle Assay

Cells were plated in 6-well plates for cell cycle assay using the cell cycle detection kit (KeyGen, Nanjing, China). Briefly, the cells were treated with 0, 0.1, 0.5, and 1 μmol/L DON for 24 h. Following three consecutive washes with ice-cold PBS and trypsinization, the harvested cells were fixed with 500 µL of chilled 70% ethanol and incubated overnight at 4 °C. After washing with PBS, the cells were stained with a propidium iodide (PI)/RNase A staining solution according to the manufacturer’s protocol, followed by flow cytometric analysis using a Beckman Coulter FACSCalibur system (Miami, FL, USA).

### 2.7. Cell Apoptosis Analysis

Apoptosis quantification was conducted with the Annexin V-FITC Apoptosis Detection Kit (KeyGen, Nanjing, China) following standardized flow cytometry protocols. Briefly, cells were treated with 0, 0.1, 0.5, and 1 μmol/L DON for 24 h. Following sequential washing and trypsinization, the cellular suspensions were centrifuged at 1200× *g* for 10 min and subsequently re-suspended in 500 µL of binding buffer. Annexin V-FITC/PI dual staining was performed according to the manufacturer’s protocol, followed by flow cytometric analysis using a FACSCalibur system (Beckman Coulter, Miami, FL, USA).

### 2.8. Quantitative Real-Time PCR (qRT-PCR) Assay

Cells were plated in 6-well plates and treated with either DON (0.5 μmol/L) alone, ABPE (200 μg/mL) alone, or co-treated with DON and ABPE for 24 h. Total RNA of bovine GCs was extracted using the FastPure^®^ Cell/Tissue Total RNA Isolation Kit V2 (Vazyme, Nanjing, China) according to the manufacturer’s protocol. RNA concentrations were quantified using a NanoDrop^TM^ 2000 spectrophotometer (Thermo Fisher Scientific, Waltham, MA, USA). Following the instructions of the HiScript ^®^ II Q RT SuperMix for qPCR (+gDNA wiper) Kit (Vazyme, Nanjing, China), 1 μg of total RNA was utilized to generate cDNA. Specific primers were designed using Primer Premier 5.0 and are listed in Table A1. qRT-PCR was conducted with ChamQ Universal SYBR qPCR Master Mix (Vazyme, Nanjing, China) on a CFX384 real-time PCR detection system (Bio-Rad, Hercules, CA, USA). The total reaction solution of 10 μL included 5 μL of SYBR Green Mix, 0.2 μL each of forward and reverse primers, 1 μL of cDNA template, and 3.6 μL of nuclease-free H_2_O. The thermal cycling parameters were set as follows: initial denaturation at 95 °C for 2 min; 40 cycles of 95 °C for 30 s, 60 °C for 30 s, and 72 °C for 30 s; followed by melt curve analysis from 65 °C to 95 °C in 0.5 °C increments every 5 s. Gene expression quantification was normalized against *GAPDH* using the 2^−ΔΔCT^ method.

### 2.9. Western Blotting Assay

Cells were plated in 6-well plates and treated with either DON (0.5 μmol/L) alone, ABPE (200 μg/mL) alone, or co-treated with DON and ABPE for 24 h. Cells underwent three washes with ice-cold PBS, followed by lysis with 110 μL of RIPA buffer (Servicebio, Wuhan, China) supplemented with protease/phosphatase inhibitors (1% PMSF, 2% cocktail, 1% phosphatase inhibitor A, and 1% phosphatase inhibitor B). The lysate was incubated on ice for 30 min before total protein collection through centrifugation at 13,400× *g* for 10 min at 4 °C. The proteins were denatured by boiling in protein loading buffer for 5 min and separated using 10% sodium dodecyl sulfate–polyacrylamide gel electrophoresis (SDS-PAGE), then transferred onto the 0.22 µm polyvinylidene fluoride (PVDF) membranes (Epizyme, Shanghai, China). Afterward, the membranes were blocked with 5% skim milk (Biosharp, Hefei, China) for 2 h at RT. The membranes were incubated overnight at 4 °C with primary antibodies against BAX, PCNA, StAR, CYP19A1, CYP11A1, and GAPDH. The details of these antibodies are listed in Table A2. Next, the membranes were washed three times with Tris Buffered Saline-Tween (TBST) and incubated with horseradish peroxidase (HRP)-conjugated secondary antibody (Table A2) for 2 h at RT. After washing with TBST three times, the membranes were developed using enhanced chemiluminescence (ECL) solution (Biosharp, Hefei, China). Finally, the quantification of gray values for Western blot bands was conducted using ImageJ analysis software (v 1.8.0, NIH).

### 2.10. Steroid Hormone Detection

Cells were plated in 6-well plates and treated with DON alone, ABPE alone, or a combination of DON and ABPE for 24 h. Meanwhile, the culture medium was supplemented with 10 μmol/L forskolin (FSK) and 200 nM androstenedione (both from Sigma-Aldrich, St. Louis, MO, USA) to induce steroidogenesis. According to the manufacturer’s protocols, the supernatants were collected for steroid hormone detection using the Bovine Progesterone (PROG) ELISA kit (CUSABIO, Wuhan, China) and the Bovine Estradiol (E2) ELISA kit (CUSABIO, Wuhan, China). Finally, a microplate reader was employed to quantify optical density values at the 450 nm wavelength. The concentrations of progesterone (P4) and estradiol (E2) were calculated based on the corresponding standard curves and normalized to the protein concentration of each sample, expressed in ng/μg protein and pg/μg protein, respectively. The ranges of the standard curves were 0.15–70 ng/mL for P4 and 40–1000 pg/mL for E2. The intra- and inter-assay coefficients of variation were both less than 15.0% for P4 and E2.

### 2.11. Reactive Oxygen Species (ROS) Assay

Cells were plated in 6-well plates and treated with either DON alone, ABPE alone, or a combination of DON and ABPE for 24 h. The intracellular ROS content was measured using a ROS assay kit (Beyotime, Beijing, China). Briefly, the cells were trypsinized and collected into 1.5 mL tubes, then washed three times with FBS-free medium, and added appropriate DCFH-DA diluted in FBS-free medium at a ratio of 1:1000 and incubated at 37 °C for 30 min. Finally, the cells were washed with PBS to remove any excess dye, and total intracellular ROS levels were quantified using a microplate reader (excitation: 488 nm; emission: 525 nm).

### 2.12. Malondialdehyde (MDA) Assay

Cells were plated in 6-well plates and treated with either DON alone, ABPE alone, or a combination of DON and ABPE for 24 h. MDA levels were determined with a cell malondialdehyde (MDA) assay kit (Jiancheng, Nanjing, China). Briefly, the cells were scraped using a cell scraper and collected into tubes after the removal of the cell culture medium. Next, the cells were broken and transferred into 1.5 mL tubes. Afterward, three types of tubes were prepared: blank tubes, standard tubes, and test tubes. After mixing the solutions, the samples were incubated in a 95 °C water bath for 40 min, cooled under running water, and then centrifuged at 1500× *g* for 10 min. After scanning the empty 96-well plate at 530 nm, 0.25 mL of each reaction solution was then added to the plate, and a microplate reader was employed to quantify optical density values at the 530 nm wavelength.

### 2.13. Total Antioxidant Capacity (T-AOC) Assay

Cells were plated in 6-well plates and subsequently co-treated with DON and ABPE for 24 h. Total antioxidant capacity (T-AOC) was quantified using a specialized T-AOC assay kit (Jiancheng, Nanjing, China). Briefly, the cells were scraped with a cell scraper and disrupted in 200 μL of cold PBS. The cells were then centrifuged at 4 °C at 13,550× *g* for 5 min, and the supernatant was collected. Afterward, three types of tubes were prepared: blank tubes, standard tubes, and test tubes. After mixing the solutions, the samples were incubated for 6 min at RT. Finally, a microplate reader was employed to quantify optical density values at the 405 nm wavelength.

### 2.14. RNA-Seq Analysis

Cells were plated in 6-well plates and treated with 0 and 0.5 μmol/L DON for 24 h. The total RNA of cells was extracted using a FastPure^®^ Cell/Tissue Total RNA Isolation Kit, and the samples were subsequently sent to Novogene Co. Ltd. (Beijing, China). RNA quantification and quality assessment were conducted using a NanoDrop^TM^ 2000 spectrophotometer (Thermo Fisher Scientific, Waltham, MA, USA). RNA integrity was evaluated using the Bioanalyzer 5400 system (Agilent Technologies, Santa Clara, CA, USA) to obtain RNA integrity numbers (RIN). RNA sequencing libraries were prepared and analyzed on the NovaSeq 6000 platform (Illumina, San Diego, CA, USA), followed by sequence alignment and annotation against the Bos taurus reference genome (ARS-UCD1.2 assembly).

The differentially expressed genes (DEGs) were identified using the thresholds of adjusted p-value (padj) < 0.05 and |fold change| > 1.5. Gene Ontology (GO) and Kyoto Encyclopedia of Genes and Genomes (KEGG) enrichment analyses were conducted using the clusterProfiler R package (Version 4.0.0), with results deemed statistically significant at padj < 0.05.

### 2.15. Statistical Analysis

All experimental procedures were performed in triplicate biological replicates, with quantitative results expressed as mean ± SEM. Graphpad Prism 6.0 software was used for data analysis, and the differences were assessed using Student’s *t*-test or one-way ANOVA analyses. Statistical significance was established at *p* < 0.05, with significance levels denoted by asterisks (* for *p* < 0.05; ** for *p* < 0.01).

## 3. Results

### 3.1. DON Suppresses Cell Proliferation and Induces Apoptosis of bGCs

**Figure 1:**
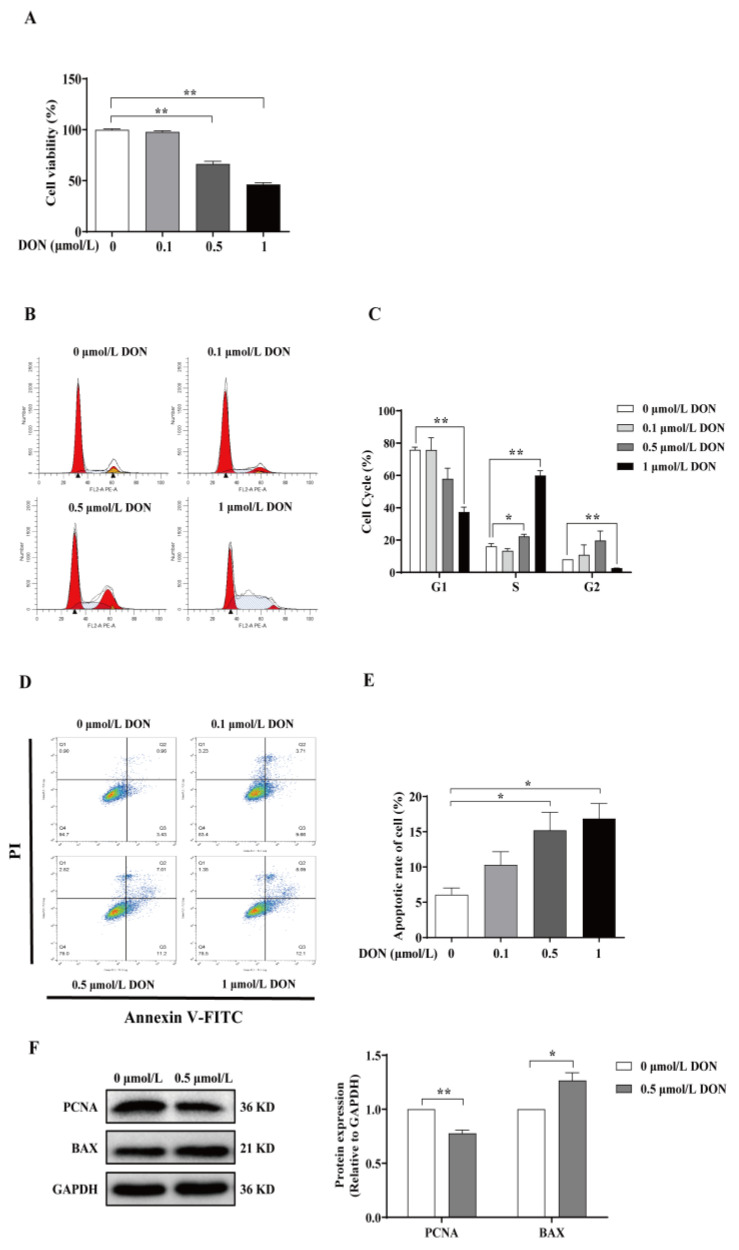
The cellular toxicity of DON on bovine granulosa cells. (**A**–**E**) Cells were treated with various concentrations of DON (0, 0.1, 0.5, and 1 μmol/L) for 24 h. The effects of DON on cell viability (**A**), cell cycle (**B**,**C**), and apoptosis (**D**,**E**) were detected. Scatter plots of Annexin V-FITC/PI dual staining were analyzed in quadrants. Q1: necrotic cells, Q2: late apoptotic cells, Q3: early apoptotic cells, and Q4: viable cells. (**F**) Cells were treated with 0.5 μmol/L DON for 24 h and conducted Western blot analysis. The quantitative results were presented as fold changes in the ratios of PCNA/GAPDH and BAX/GAPDH relative to the control group. * *p* < 0.05, ** *p* < 0.01.

Bovine GCs were treated with various concentrations of DON for 24 h to evaluate its cytotoxic effects. Cell proliferation and apoptosis were subsequently detected. CCK-8 results showed that 0.5 and 1 μmol/L DON significantly decreased the viability of bovine GCs by 33.53% and 53.65%, respectively (Figure 1A, *p* < 0.01). In contrast, treatment with 0.1 μmol/L DON did not have a significant effect on the viability of bovine GCs (Figure 1A, *p* > 0.05). Furthermore, flow cytometry analysis revealed that 1 μmol/L DON significantly reduced the proportion of cells in the G1 and G2 phases (Figure 1B,C, *p* < 0.01), while increasing the proportion of cells in the S phase (Figure 1B,C, *p* < 0.01), indicating that DON treatment can induce the cell cycle arrest in GCs. Compared with the control group, 0.5 and 1 μmol/L DON exposure significantly increased cell apoptosis (*p* < 0.05, Figure 1D,E), with apoptosis rates of 15.21% and 16.87%, respectively. In addition, Western blot analysis revealed that 0.5 μmol/L DON exposure significantly decreased the expression of the proliferative protein marker PCNA (*p* < 0.01) and increased the expression of the pro-apoptotic protein BAX (*p* < 0.05, Figure 1F).

### 3.2. DON Alters Gene Expression Patterns of bGCs

**Figure 2:**
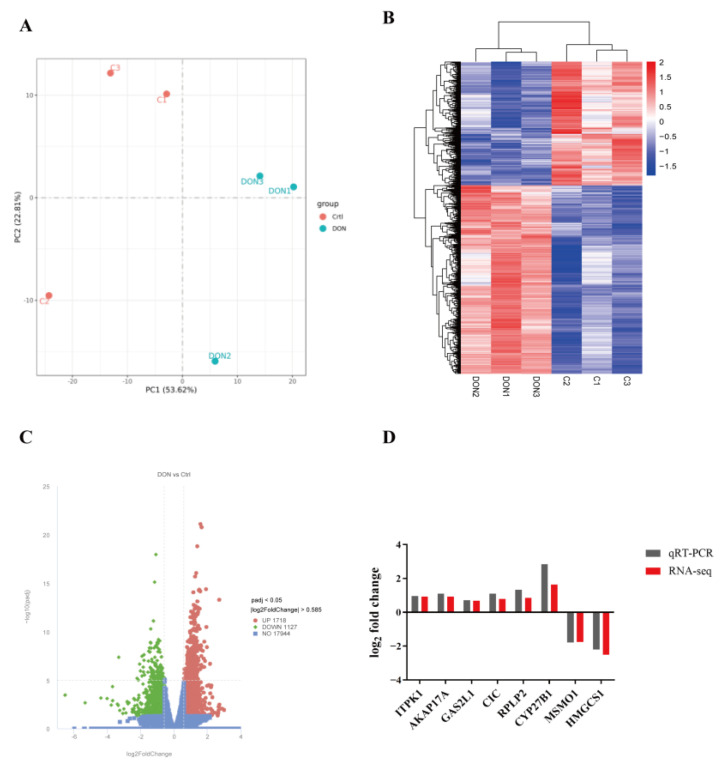
Transcriptome analysis of bovine GCs after DON exposure. (**A**) The principal component analysis (PCA) of sample relationships. (**B**) The heatmap of DEGs in bovine GCs between the control group and the DON exposure group. (**C**) Volcano plot of DEGs in bovine GCs between the control group and the DON exposure group. Red and green dots represent genes with significantly differences (|fold change| > 1.5 and padj < 0.05), whereas blue dots represent genes with no significant differences. (**D**) Transcriptional validation of eight randomly selected DEGs was performed using qRT-PCR analysis.

To better understand the molecular mechanisms underlying DON-induced cytotoxicity in bovine GCs, we further examined gene expression profiles by performing RNA-seq analysis. Principal component analysis (PCA) showed a clear separation between the DON exposure group and the control group (Figure 2A), which was corroborated by the heat map of correlation between samples based on gene expression levels (Figure 2B). The RNA-seq analysis identified a total of 2845 differentially expressed genes (DEGs) between the DON treatment group and the control group, including 1718 up-regulated genes (Figure 2C, red dots) and 1127 down-regulated genes (Figure 2C, green dots). In addition, eight DEGs were randomly selected for qRT-PCR analysis, and the qRT-PCR results were consistent with the RNA-seq data (Figure 2D), indicating that the RNA-seq results were reliable in our study.

**Figure 3:**
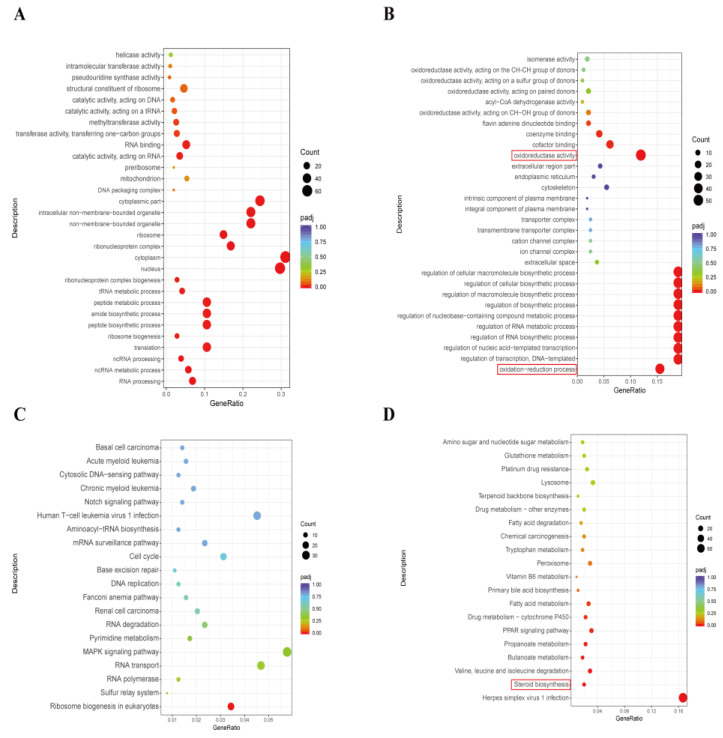
GO and KEGG analyses of the DEGs in bovine GCs after DON exposure. (**A**) Scatter plot of the most enriched GO terms for the up-regulated DEGs. (**B**) Scatter plot of the most enriched GO terms for the down-regulated DEGs. (**C**) Scatter plot showing the top 20 enriched KEGG pathways for the up-regulated DEGs. (**D**) Scatter plot showing the top 20 enriched KEGG pathways for the down-regulated DEGs. The size of the dots represents the number of enriched genes, with larger dots indicating a greater magnitude of enrichment. The color of the dots reflects the levels of statistical significance, represented by the adjusted *p*-value (padj).

The up-regulated and down-regulated DEGs were subsequently analyzed using GO and KEGG analyses. The up-regulated DEGs were primarily enriched in GO terms related to translation, cytoplasm, and ribosomal biogenesis (Figure 3A). Meanwhile, GO functional enrichment analysis of the down-regulated DEGs was associated with oxidative stress process, including the oxidation-reduction process, oxidoreductase activity, and regulation of cellular biosynthetic process (Figure 3B). The scatter plots of KEGG pathway enrichment analysis, based on the up-regulated and down-regulated DEGs, showed that the significantly enriched pathways mainly included ribosome biogenesis and steroid biosynthesis (Figure 3C,D). These findings indicate that DON exposure may impact steroid biosynthesis and induce cellular oxidative stress in bovine granulosa cells.

### 3.3. DON Inhibits Steroid Hormone Synthesis and Induces Oxidative Stress in bGCs

**Figure 4:**
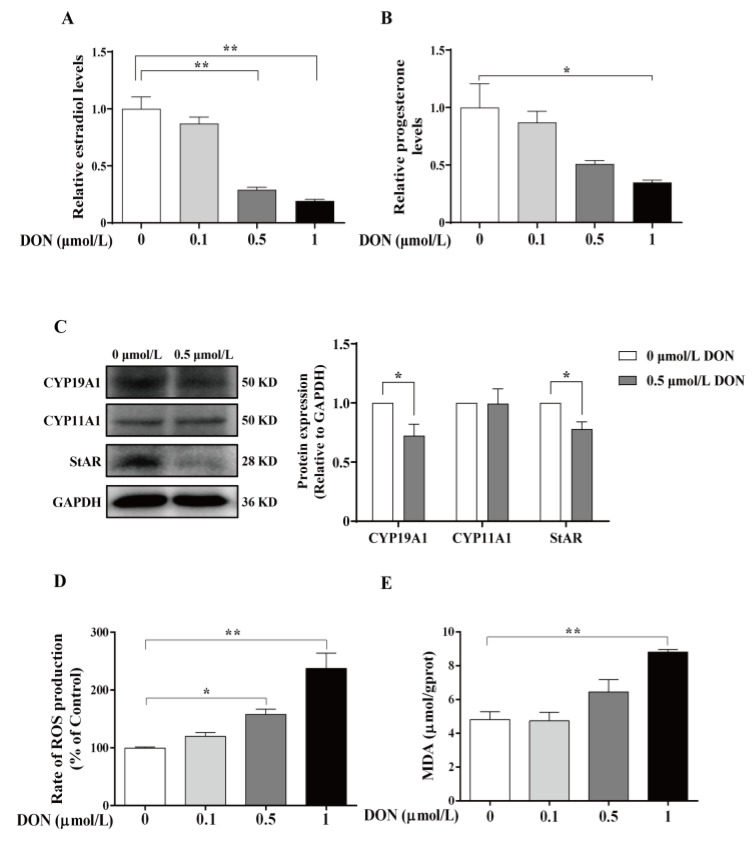
The toxic effects of DON on steroid hormones and oxidation reduction in bGCs. (**A**,**B**) Cells were treated with various concentrations of DON (0, 0.1, 0.5, and 1 μmol/L) for 24 h, after which the levels of estradiol and progesterone in the supernatant were measured by ELISA. Data were normalized based on the protein concentration of each sample well and expressed as fold changes relative to the control. (**C**) Cells were treated with 0.5 μmol/L DON for 24 h and Western blot analysis was conducted. The quantitative results were presented as fold changes in the ratios of CYP19A1/GAPDH, CYP11A1/GAPDH, and StAR/GAPDH relative to the control group. (**D**,**E**) Cells were treated with various concentrations of DON (0, 0.1, 0.5, and 1 μmol/L) for 24 h, and the production of ROS and MDA levels were measured. * *p* < 0.05, ** *p* < 0.01.

Because transcriptome analysis data showed that DON exposure altered the steroid biosynthesis pathway and the cellular oxidative stress process, we further assessed the levels of estrogen and progesterone in the supernatant of bovine GCs. Compared with the control group, 1 μmol/L DON exposure significantly decreased the secretion of estrogen (*p* < 0.01, Figure 4A) and progesterone (*p* < 0.05, Figure 4B). Moreover, 0.5 μmol/L DON exposure significantly reduced estrogen production (*p* < 0.01, Figure 4A), but had no significant effect on progesterone (*p* > 0.05, Figure 4B). Western blot analysis indicated that 0.5 μmol/L DON exposure decreased the expression of StAR and CYP19A1 (*p* < 0.05, Figure 4C), but did not affect the expression of CYP11A1. These findings demonstrate that DON may inhibit steroidogenesis by downregulating the expression of steroidogenic-related genes.

Meanwhile, we assessed the levels of reactive oxygen species (ROS) and malondialdehyde (MDA) in bovine GCs. The results showed that, with the increase in DON concentration, the levels of ROS and MDA in granulosa cells rose in a dose-dependent manner (Figure 4D,E). Notably, the group treated with 1 μmol/L DON exhibited a significant increase in the levels of ROS and MDA in granulosa cells (*p* < 0.01). These findings indicate that DON can induce oxidative stress by increasing the levels of ROS and MDA in bovine granulosa cells.

### 3.4. ABPE Alleviates the Decrease of Cell Viability and Steroid Hormones Caused by DON in bGCs

**Figure 5:**
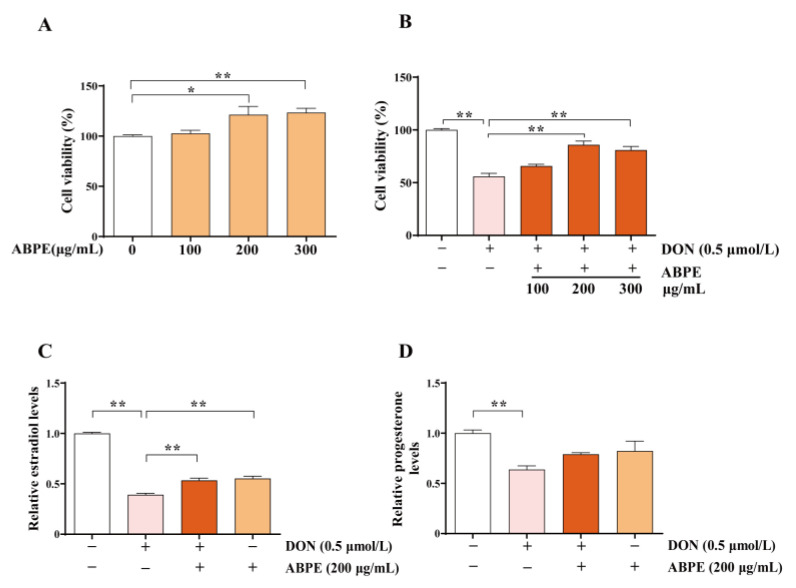
The protective effects of ABPE on the decrease of cell viability and steroid hormone levels induced by DON in bGCs. (**A**) Cell viability was assessed after treating the cells with various concentrations of ABPE (0, 100, 200, and 300 μg/mL) for 24 h. (**B**) Cells were treated with 0.5 μmol/L DON and different concentrations of ABPE (0, 100, 200, and 300 μg/mL) for 24 h, and cell viability was measured. (**C**,**D**) Cells were treated with 0.5 μmol/L DON and 200 μg/mL of ABPE for 24 h. The levels of estradiol (**C**) and progesterone (**D**) in the supernatant were measured by ELISA. Data were normalized based on the protein concentration of each sample well and expressed as fold changes relative to the control. * *p* < 0.05, ** *p* < 0.01.

Considering the well-documented multiple functions of bee pollen (BP), including its antimicrobial, immunostimulating, and antioxidant effects [16], we further evaluated the alleviative effect of apricot bee pollen ethanol extract (ABPE) on the cellular toxicity of DON in bovine GCs. The average concentration of phenolic compounds in ABPE was 9.21 ± 0.28 mg GAE/g. Following treatment of bovine GCs with 100, 200, and 300 μg/mL ABPE for 24 h, the results showed that ABPE significantly increased the viability of bovine GCs (*p* < 0.05, Figure 5A). Regarding co-treatment with 100, 200, and 300 μg/mL ABPE and 0.5 μmol/L DON for 24 h, both 200 μg/mL and 300 μg/mL ABPE significantly alleviated the toxic effects of DON on the viability of bovine GCs (*p* < 0.01, Figure 5B). In addition, ELISA results showed that ABPE significantly relieved the toxic effect of DON on estrogen secretion in bovine GCs (*p* < 0.01, Figure 5C); however, ABPE did not exhibit a significant effect on progesterone secretion (Figure 5D).

### 3.5. ABPE Alleviates Oxidative Stress Induced by DON in bGCs

**Figure 6:**
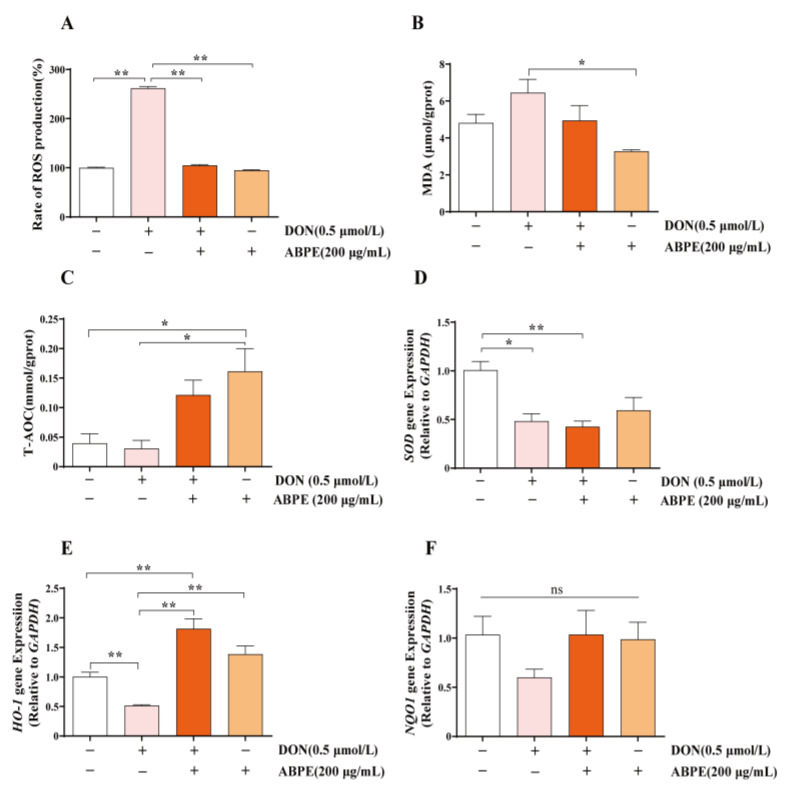
The protective effects of ABPE against oxidative stress induced by DON in bGCs. Cells were treated with 0.5 μmol/L DON and 200 μg/mL ABPE for 24 h, and the production of ROS (**A**), MDA levels (**B**), and T-AOC levels (**C**) were measured. The relative mRNA expression levels of *SOD* (**D**), *HO-1* (**E**), and *NQO1* (**F**) were detected by qRT-PCR with *GAPDH* normalization. * *p* < 0.05, ** *p* < 0.01, ns indicates no significance.

After co-treatment with 200 μg/mL ABPE and 0.5 μmol/L DON for 24 h, the intracellular ROS and MDA levels were measured. The results showed that 200 μg/mL ABPE significantly reduced the ROS levels induced by DON (Figure 6A, *p* < 0.01); however, it did not significantly alleviate the DON-induced increase in MDA levels (Figure 6B). Notably, ABPE treatment alone group exhibited a significant increase in T-AOC activity compared with the control group (*p* < 0.05, Figure 6C). Although T-AOC activity showed an increasing tendency in the ABPE and DON co-treatment group, this increase was not statistically significant when compared with the DON-only group. Furthermore, qRT-PCR results indicated that, compared with the control group, the mRNA level of the antioxidant-related gene *superoxide dismutase* (*SOD*) was significantly decreased in the 0.5 μmol/L DON treatment group (*p* < 0.05, Figure 6D). Although ABPE did not alleviate the reduction in *SOD* mRNA levels caused by DON (Figure 6D), it significantly increased the expression of *heme oxygenase-1* (*HO-1*, *p* < 0.01, Figure 6E). The expression of the antioxidant gene *NAD(P)H quinone dehydrogenase 1* (*NQO1*) remained unaffected by either DON or ABPE treatment (Figure 6F). These results suggest that ABPE can alleviate DON-induced oxidative stress by upregulating the expression of *HO-1*.

## 4. Discussion

In cattle, DON has been found to inhibit cell proliferation and induce apoptosis in different kinds of cells, such as bovine peripheral blood mononuclear cells [24], mammary epithelial cells [22,25], renal epithelial cells [26], and granulosa cells [11]. In this study, we demonstrated that DON exposure reduced the proliferation of bovine GCs with a significant downregulation of PCNA, and further revealed DON exposure can induce cell cycle arrest at the S phase. Meanwhile, DON exposure increased cell apoptosis, along with a significant upregulation of BAX. Our results are consistent with previous studies regarding the effects of DON on the activity and apoptosis of GCs in cattle [11], pigs [27], and mice [10]. In contrast, Medvedova et al. [28] observed that high-dose DON exposure increased PCNA expression in porcine ovarian GCs without inducing apoptosis. This discrepancy may be attributed to variations in DON concentration and different culture conditions.

To further investigate the toxicity and mechanisms of DON exposure in bovine GCs, we conducted the RNA-seq analysis. Results showed that the DEGs were enriched in the oxidation-reduction process, oxidoreductase activity, regulation of the biosynthetic process, and steroid biosynthesis, which are associated with the function of granulosa cells. Subsequently, we demonstrated that DON exposure decreased the production of estrogen and progesterone, resulting from the downregulation of CYP19A1 and StAR protein expression. This finding aligns with the observations made by Guerrero-Netro et al. [11]. In fact, DON has been recognized as an endocrine disruptor that may affect the synthesis of steroid hormones [29,30]. The observed disturbances of DON in steroid production may be mediated by its binding to ribosomes and induction of the ribotoxic stress response via p38, ERK1/2, and c-Jun [11,12]. Furthermore, we observed that DON exposure induced oxidative stress by increasing the levels of ROS and MDA in bovine GCs, which is well consistent with our RNA-seq data. Recent studies have also demonstrated that DON induces cellular oxidative damage through the generation of ROS in murine ovarian GCs [13] and human GCs [9]. It is important to note that the apoptosis induced by DON in bovine GCs may be mediated by oxidative stress, which has been demonstrated to induce apoptosis in granulosa cells through the ROS-JNK-p53 pathway [31].

Given the above toxic evidence of DON in granulosa cells, researchers aimed to develop effective strategies to mitigate the toxicity of DON. Among potential approaches, melatonin has been widely used to alleviate DON toxicity due to its well-known antioxidant effect. Fan et al. [13] demonstrated that melatonin can alleviate DON-induced oxidative stress and mitochondrial dysfunction in murine GCs. Similarly, Xue et al. [9] also revealed that melatonin can protect human GCs against DON-induced oxidative damage by suppressing FOXO1 and ER stress. In addition to melatonin, resveratrol and amygdalin have been reported to alleviate DON-induced dysregulation of steroid hormones in porcine GCs [14,15]. However, there are few reports on alleviating the toxic effects of DON in bovine ovarian GCs. Here, we demonstrated that ABPE enhances the activity of bovine ovarian GCs and significantly alleviates the toxic effects of DON exposure on bovine GCs. We first observed that ABPE increased the viability of bovine granulosa cells in a dose-dependent manner, which extended findings from a previous study in porcine granulosa cells showing that bee pollen had no effect on PCNA and caspase-3 expression in porcine GCs [32]. The obvious promoting proliferation effect of ABPE observed in this study may be attributed to the use of the ethanol extract method. Importantly, ABPE was found to efficiently alleviate the reduction in cell viability induced by DON.

According to in vitro and in vivo experiments, bee pollen exhibits significant antioxidant activity and is recognized as a free radical scavenger and lipid peroxidation inhibitor. The antioxidant activity of bee pollen is primarily due to its phenolic compounds [33], which have been quantified in our ABPE samples. ABPE reduced the levels of ROS induced by DON in bovine ovarian GCs, while also upregulating the mRNA expression of the antioxidant-related gene *HO-1*. The excessive production of ROS is considered a major contributor to oxidative stress, which can initiate the process of lipid peroxidation in the lipid membranes, resulting in DNA damage and cell apoptosis [34,35]. Heme oxygenase-1 (HO-1) is an antioxidant factor regulated by the nuclear factor erythroid 2-related factor 2 (Nrf2) [36], and the increase in *HO-1* mRNA expression following the addition of ABPE indicates an enhancement in antioxidant capacity.

In addition, a previous study showed that bee pollen in combination with metformin increases serum estrogen levels in rats [37], which may be attributed to its estrogenic potential. Similarly, our research revealed that ABPE can enhance estrogen secretion in bovine GCs under DON exposure. Furthermore, bee pollen has been shown to exhibit a protective effect against the immunotoxic effects induced by the consumption of aflatoxins in rats [19]. Therefore, our study further extends the understanding of the functions of bee pollen by highlighting its protective effects against DON-induced cell viability reduction, estrogen disorder, and oxidative stress.

## 5. Conclusions

Our results demonstrate that DON induces apoptosis, estrogen dysfunction, and oxidative stress in bovine granulosa cells. Importantly, we provide new insights into the protective effects of bee pollen against DON-induced cell viability reduction, estrogen disorder, and oxidative stress, indicating that bee pollen is a promising natural agent for preventing DON-induced reproductive toxicity.

## Data Availability

The data used to support the findings of this study are available from the corresponding author upon request.

## Appendix A

**Table A1:** Primer information.

Genes	Sequence (5′-3′)	Length (bp)	Accession No.
*GAPDH*	F: GGTCACCAGGGCTGCTTTTA	222	NM_001034034.2
	R: CCAGCATCACCCCACTTGAT		
*SOD*	F: GCTGGAAGCCATCAAACG	107	NM_201527.2
	R: TGAAGCCGAGCCAACCC		
*NQO1*	F: TTCAATCCCGTCATCTCC	215	NM_001034535.1
	R: TCAAACCAGCCTTTCAGG		
*HO-1*	F: CTGGTGATGGCGTCTTTGT	215	NM_001014912.1
	R: TCCTGGAGTCGCTGAACAT		
*MSMO1*	F: TTTGCCTGACAATCCT	140	NM_001098863.1
	R: ATCCAGGTAAACAGAACA		
*CYP27B1*	F: TCGGCTCGGTGTTCGTG	171	NM_001192284.1
	R: CAGACTGGTTCCTCATGGCTAC		
*ITPK1*	F: CTGCTCGCCACCCTTCA	114	NM_001192489.3
	R: CCATGAGCCACTCTGGTTTT		
*RPLP2*	F: GGCTCAACAAGGTCATCAGTG	158	NM_174788.4
	R: TGCGGCTGGAGCAGAAC		
*AKAP17A*	F: GGAGAACAAGAGCCTCGTCAAGT	147	XM_024990316.2
	R: CGGAAGAAGGAGTCCCAGTCG		
*GAS2L1*	F: CCCAGTCCAGAGTTGAGCACA	250	NM_001083698.1
	R: GGACGCTGAGGGACGAAGA		
*CIC*	F: TTTTGAGTTTGACGAGTGCGAAGC	151	XM_015458193.3
	R: AGGGCGGAAGCCGAAGAGTG		
*HMGCS1*	F: GAGGAGTCTGGGAATACG	129	NM_001206578.1
	R: TACCAGGGCATACCGT		

**Table A2:** Antibodies used in this study.

Antibodies	Dilution Ratio	Supplier	RRID	Cat. No	MW (KD)
BAX	1:4000	ABmart, China	AB_2910262	T40051	21
PCNA	1:1000	Proteintech, USA	AB_2160330	10205-2-AP	36–38
StAR	1:1000	ABclonal, China	AB_2772418	A16432	28
CYP19A1	1:500	Cell Signaling, USA	AB_2630344	14528s	50
CYP11A1	1:1000	ABclonal, China	AB_2763764	A1713	50
GAPDH	1:5000	ABclonal, China	AB_2736879	AC002	36
HRP Goat Anti-Mouse IgG (H + L)	1:8000	ABclonal, China	AB_2769851	AS003	
HRP Goat Anti-Rabbit IgG (H + L)	1:8000	ABclonal, China	AB_2769854	AS014	
